# Details of the female figure in profile according to Leonardo da Vinci

**DOI:** 10.1016/j.jpra.2026.03.018

**Published:** 2026-03-11

**Authors:** Edoardo Raposio, Elisa Bertulla

**Affiliations:** a Plastic Surgery Chair, Department of Surgical Sciences and Integrated Diagnostics (DISC), University of Genova, Largo Benedetto XV, n.6, Genova 16132, Italy; b Plastic and Reconstructive Surgery Division, IRCCS Azienda Ospedaliera Metropolitana, Largo Rosanna Benzi, n.10, Genova 16132, Italy

**Keywords:** Navel, Female aesthetics, Female abdomen, Female beauty

Leonardo da Vinci (1452–1519) is widely regarded as one of the most brilliant minds of Western civilization. An Italian polymath of the High Renaissance, he was active as a painter, draughtsman, engineer, scientist, theorist, sculptor, and architect. By combining extraordinary artistic mastery with observational and scientific studies that were highly unconventional for his time, Leonardo produced numerous masterpieces that continue to endure, including the Mona Lisa and The Last Supper.

In recent years, our group has focused on studying the rules and geometries specific to the female figure,^1^, ^2^, ^3^, ^4^, ^5^, ^6^ with the aim of identifying guidelines that may help define principles of proportion and ideal female beauty. Through an analysis of Leonardo’s anatomical drawings, we observed that in one particular plate (Figure 1), depicting the female figure in profile, the navel is ideally positioned along an imaginary horizontal line connecting it to the apex of the iliac crest and the point of maximum concavity of the dorsal profile. Prompted by this observation, we further noted that this characteristic appears to be common among many female figures in the entertainment and fashion industries who are widely regarded as aesthetically pleasing. This feature could be quantitatively assessed in future studies aimed at investigating population-based aesthetic preferences, particularly with respect to the spatial relationship among the three anatomical landmarks considered (navel, iliac crest apex, and dorsal concavity). Although this is only an isolated observation and therefore devoid of any scientific or experimental basis, it can serve as a starting point for subsequent studies that may validate or not these hypotheses.

**Figure 1:**
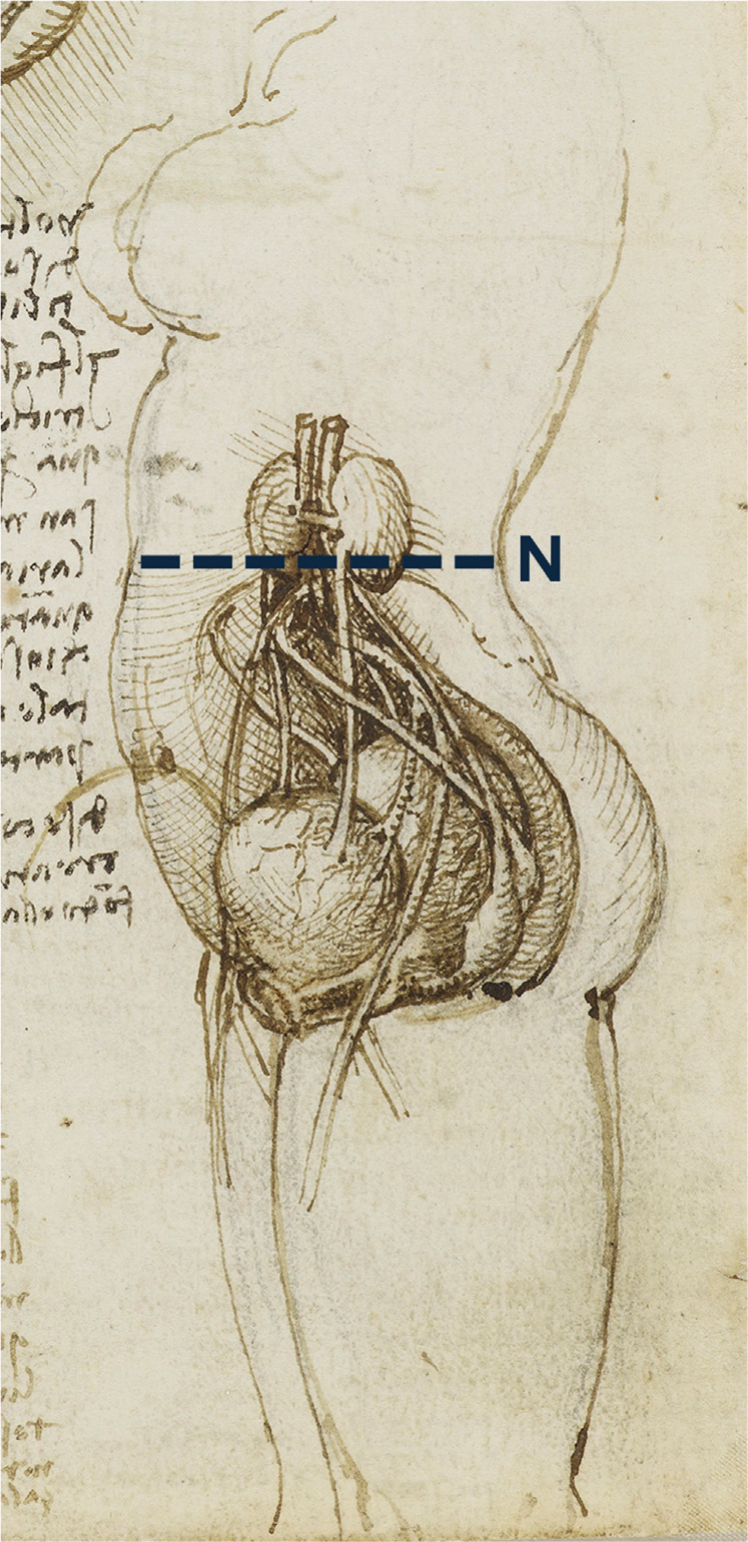
Left lateral view of a standing female figure. The dashed blue line (N) ideally connects the navel, the apex of the iliac crest, and the point of maximum concavity of the posterior profile. Folio from Leonardo da Vinci’s *Anatomical Manuscript B*, verso, detail. Reproduced courtesy of Royal Collection Enterprises Limited © 2025 | Royal Collection Trust.

## Funding

None.

## Declaration of competing interest

None.
